# Six-Month Local Control Rates and Immune Responses After Pulsed Electric Field Ablation in Metastatic Cancer

**DOI:** 10.3390/cancers17213495

**Published:** 2025-10-30

**Authors:** Alicia Moreno-Gonzalez, Ebtesam H. O. Nafie, Chiara Pastori, Joseph Mammarappallil, Partha Seshaiah, Maria B. Plentl, Beryl A. Hatton, Robert E. Neal, Michael A. Pritchett, Janani S. Reisenauer, Sebastian Fernandez-Bussy, David DiBardino, Bradley B. Pua, William S. Krimsky

**Affiliations:** 1 Galvanize Therapeutics Inc., Redwood City, CA 94065, USA; 2 Duke Cancer Center, Durham, NC 27710, USA; 3 Firsthealth of the Carolinas, Pinehurst Medical Clinic, Pinehurst, NC 28374, USA; 4 Mayo Clinic, Rochester, MN 55905, USA; 5 Mayo Clinic, Jacksonville, FL 32224, USA; 6 Division of Pulmonary, Allergy and Critical Care, Department of Medicine, University of Pennsylvania, Philadelphia, PA 19104, USA; 7 New York Presbyterian Hospital, Weill Cornell Medicine, New York, NY 10065, USA

**Keywords:** ablation, immunological changes, lung cancer, metastatic cancer, pulsed electric fields

## Abstract

**Simple Summary:**

This work presents preliminary data for the Aliya System, a specialized form of ablation technology not dependent on thermal processes, with 510(k) clearance for the surgical ablation of soft tissue, evaluating local efficacy for bronchoscopic and percutaneous ablation of lung lesions. The data support that this form of ablation can achieve acceptable 6-month local control rates without impact on subsequent standard-of-care therapy in Stage IV cancer patients. This study builds on earlier findings that demonstrated the safety of this form of ablation in the lungs, including bilateral procedures, supporting its integration into clinical practice for ablating focal lesions. Importantly, this form of ablation demonstrated systemic immune activation, suggesting both cellular and humoral immune activation following ablation. The data suggests a potential opportunity to initiate patient care at the time of biopsy along with the possibility of inducing a systemic immune response.

**Abstract:**

**Background**: The AFFINITY trial (NCT05890872) is a prospective, non-randomized, open-label, single-arm study evaluating the safety, immunological impact, and preliminary efficacy of Aliya pulsed electric field ablation in patients with solid tumors. Thirty-one patients were enrolled; thirty received lung lesion ablation prior to continuation on standard-of-care treatment. This manuscript reports six-month local control outcomes and immunological response characteristics. Radiological outcomes were assessed using a modified RECIST 1.1, and immunological impact was evaluated via changes in peripheral blood immunocyte populations and detection of immunoglobulins (Ig) to tumor-associated antigens in serum post-ablation. **Methods**: Twenty-eight patients underwent radiological assessment of ablated lesions at approximately 1-, 3-, and 6-month post-ablation to evaluate local control. Peripheral blood was collected for immune monitoring using flow cytometry and to detect IgG responses to biopsy-specific and tumor-associated antigens. **Results**: At 6 months, two cohorts emerged: 12 received ablation only, and 16 received ablation plus systemic and/or focal therapies (radiotherapy or second ablation). In the ablation-only group, imaging showed local control in all ablated lesions (8/12 SD, 4/12 PR), suggesting local efficacy without systemic therapy in those patients. Immunophenotyping showed dynamic changes in circulating immune cells, including T and B cell activation. A subset also exhibited modulation of tumor antigen-specific IgG, indicating a systemic humoral response. **Conclusions**: This analysis provides preliminary evidence that this form of ablation may promote local tumor control and modulate systemic immune function. These findings support the immunogenic potential of this specialized energy and warrant further investigation. Extended 12-month data for the full cohort will be reported in a future manuscript.

## 1. Introduction

Current treatment options, including systemic therapies and local interventions, often provide limited long-term disease control in patients with metastatic cancer to the lung or advanced non-small-cell lung cancer (NSCLC) [1,2,3]. Despite advances in targeted therapies and immunotherapy, many patients with advanced malignancies experience disease progression [4], underscoring the need for novel approaches that enhance treatment efficacy while maintaining safety and tolerability.

The Aliya^®^ Pulsed Electric Field system (Galvanize Therapeutics Inc., Redwood City, CA, USA) was developed as a minimally invasive ablation approach that delivers short-duration high-voltage electric pulses through a single monopolar needle placed in the target tissue to disrupt homeostasis and induce cell death. The first-in-human (FIH) INCITE ES (NCT04732520) treat-and-resect trial in early-stage NSCLC patients demonstrated the safety and feasibility of this form of ablation [5]. This specialized energy does not rely on thermal gradients to induce cell death, enabling ablation without denaturing cellular and stromal proteins, improving the safety profile. Preclinical [6] and clinical evidence [5,7] indicates that it may also produce an immunostimulatory post-ablative tumor microenvironment, including tumor antigens and damage associated molecular patterns (DAMPs), and retaining a structural environment more conducive to infiltrating immune cells [8].

Murine tumor models have demonstrated that this form of ablation not only induces local tumor destruction, but also induces systemic immune responses [9,10,11], including a marked increase in the proportion of B-cells within the post-ablative tumor microenvironment and in circulation, correlating with significant increase in anti-gp70 IgG tumor-specific antibodies [12].

The AFFINITY study (NCT05890872) is a prospective, single-arm, non-randomized, multi-center, open-label trial designed to evaluate the safety and feasibility of this form of ablation as a cytoreductive strategy prior to the initiation of standard-of-care (SOC) therapy. This study previously demonstrated that ablation of lung lesions can be safely and effectively integrated into existing treatment paradigms [13]. This manuscript presents preliminary efficacy and exploratory data up to 6 months following ablation in the AFFINITY population, focusing on local control rates and immunological changes in patients who did not receive subsequent SOC treatment prior to their 6-month follow-up.

## 2. Materials and Methods

### 2.1. Clinical Study Design and Patient Population

This AFFINITY trial (NCT05890872) assessed the safety of ablation in patients with metastases to the lung or stage IV NSCLC. Thirty-one eligible adult participants were enrolled. Eligible patients required diagnostic lung biopsy for suspected malignancy, were not surgical candidates for curative-intent, had not previously received treatment for their targeted tumor(s), and were indicated for first-line therapy for their targeted tumor(s). Ablation was performed either bronchoscopically or percutaneously. Follow-up occurred around days 3, 10, and 30, as well as approximately 3-, 6-, 9-, and 12- months post-ablation. Adjuvant systemic therapy and/or focal therapy were administered at the discretion of the treating physician, in accordance with institutional protocols and standard clinical practice. Data collection included computed tomography (CT) imaging, adverse event (AE) monitoring, and review of relevant concomitant therapies. An independent board-certified radiologist reviewed CT images.

### 2.2. Ablation Procedure

**Figure 1:**
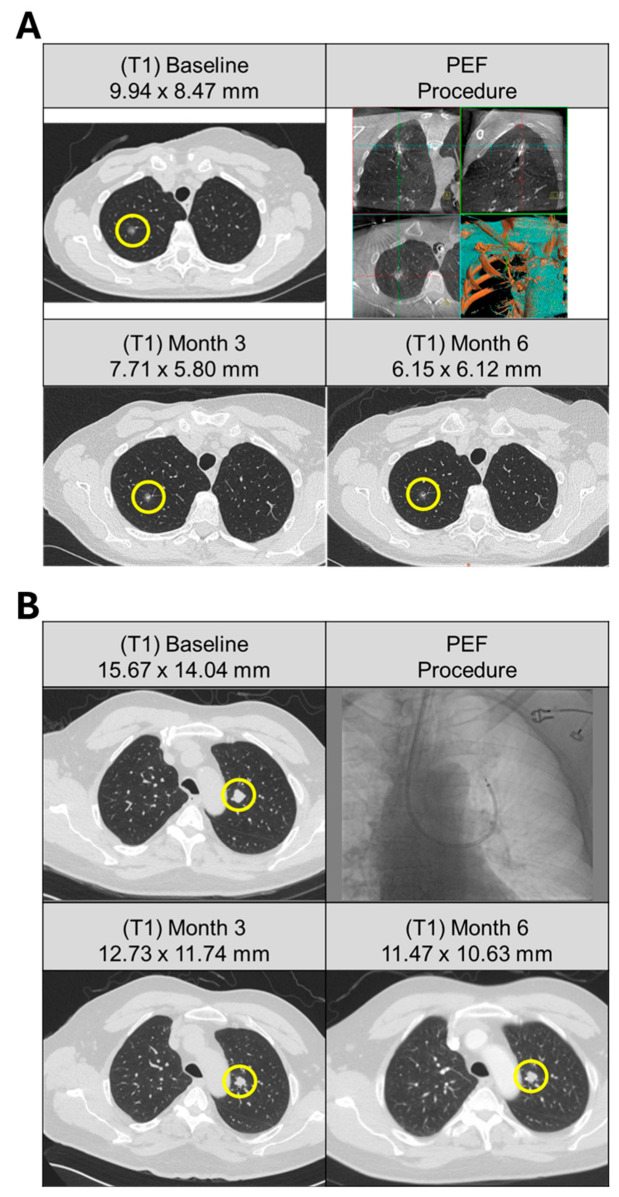
Pulmonary metastases from solitary and multifocal patients treated with bronchoscopic ablation. (**A**) Patient (A25) with history of breast and bladder cancer, presented with solitary right upper lobe lesion with no identifiable primary source. (Top left) CT scan of 9.9 mm nodule at baseline (yellow circle). Three activations were delivered to the target lesion via bronchoscopic access (top right). (Bottom row) Response of the target lesion at 3 and 6 months following ablation demonstrated a progressive decrease in size, measuring 7.7 mm and 6.2 mm, respectively. (**B**) Patient (A02) presented with multifocal solid pulmonary nodules subsequently deemed metastatic papillary thyroid carcinoma with cervical lymph node involvement. Five pulmonary lesions were identified at baseline (top left). Ablation was performed on a single-target lesion (yellow circle) in the left upper lobe using a bronchoscopic approach (top right) with nine activations. (Bottom row) Response of the target lesion at 3 and 6 months following ablation demonstrated size stability, measuring 12.7 mm and 11.5 mm, respectively.

The Aliya Generator and Aliya Ablation Device (Aliya Percutaneous Needle and Aliya Electrode) received 510(k) clearance (K212871) for the surgical ablation of soft tissue, and an investigational device (Aliya Endobronchial Needle) was evaluated in this feasibility study. The energy was delivered bronchoscopically or percutaneously to encompass as much of the total tumor burden within the lungs as clinically feasible in a single general anesthetic session (index procedure, Figure 1). Target selection included assessment of available imaging (e.g., CT, PET-CT, MRI), where the lesion was ≤5 cm in longest diameter and the anticipated ablation zone ≥ 10 mm from mediastinal, hilar, lobar structures, and diaphragm. Additional procedural details were previously described [13]. Procedural success was defined by the ability to deliver the ablation to the intended target(s). Per protocol, subsequent SOC ablation was allowed for a pre-existing tumor not treated during the index procedure, a newly identified tumor post-index procedure, or for appropriate treatment of the previously treated lesion.

### 2.3. Analysis Groups

**Figure 2:**
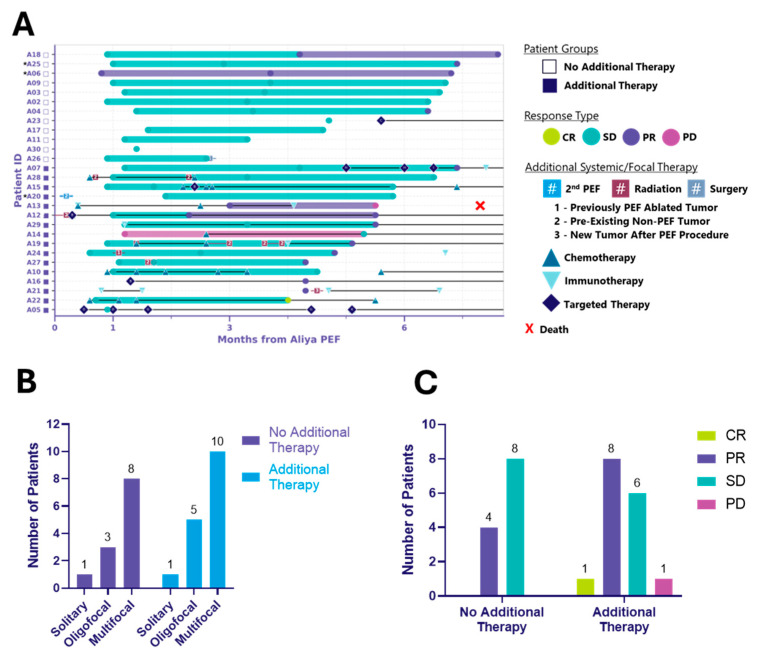
Six-month local control efficacy and clinical course following ablation. Two distinct patient cohorts emerged: those treated with ablation alone (no additional therapy) and those who received additional systemic and/or focal therapies (additional therapy). (**A**) Swimmer plot illustrating the clinical course of patients following ablation. Each bar represents the duration of response (mRECIST) of ablated tumors, with symbols indicating the time of first observed response. Additional systemic/focal therapy includes systemic therapy and procedural events initiated after the index ablation procedure and that occurred through the 6-month visit window. * Indicates patients receiving systemic therapy prior to ablation, considered to have unresolved disease activity and requiring diagnostic biopsy (see Appendix A.1). Symbols with letters or numbers indicate initiation and discontinuation of systemic or focal therapies; connected symbols denote the start and end of a given treatment. In some cases, overlapping symbols reflect multiple therapies initiated or discontinued at the same time. Black horizontal lines connecting these symbols indicate that therapy was ongoing between those time points. Black lines extending to the right edge of the graph indicate that systemic therapy was ongoing through the 6-month visit window. (**B**) Bar graph depicting the distribution of disease burden at baseline, categorized as solitary, oligofocal or multifocal disease, among patients in the no additional therapy and additional therapy groups. Disease burden was assessed prior to ablation. (**C**) Bar graph showing mRECIST-defined responses of ablated tumors at up to 6 months post-ablation, grouped by patient cohort. Responses include complete response (CR), partial response (PR), stable disease (SD), and progressive disease (PD).

**Figure A1:**
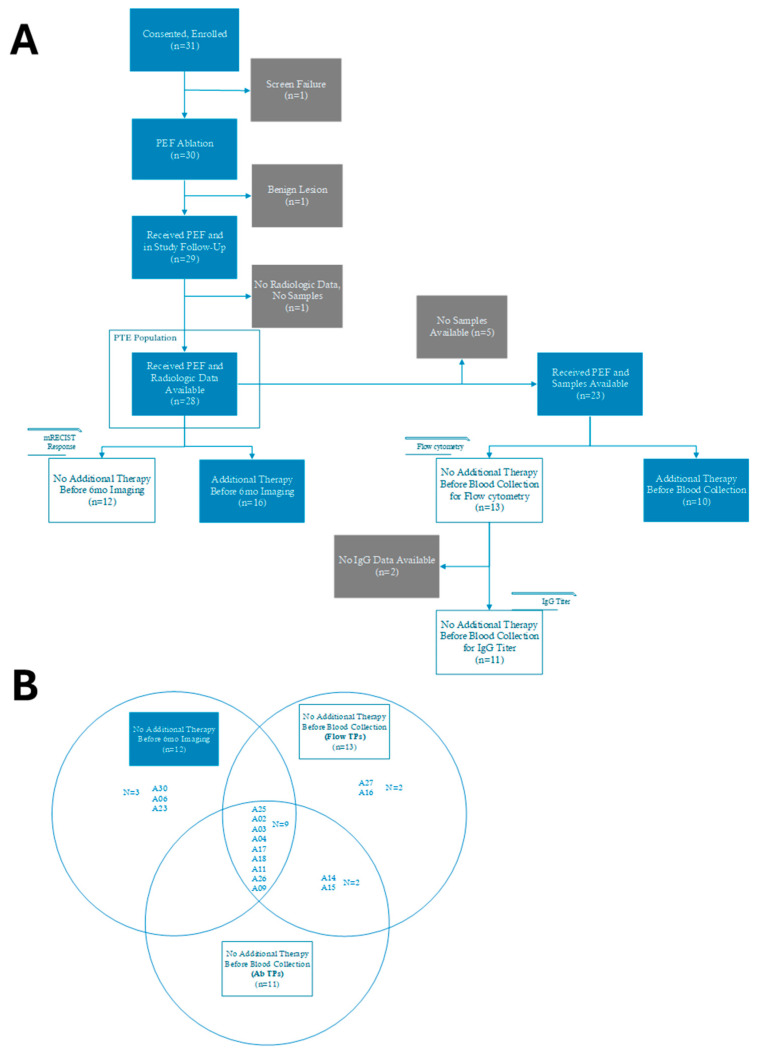
CONSORT and Venn diagrams illustrating patient exclusions and analysis cohorts. (**A**) CONSORT diagram of per-treatment evaluable (PTE) population and associated analyses. (**B**) Venn diagram showing the overlap of patient cohorts included in each of the analyses (radiographic response, flow cytometry, IgG titer).

The AFFINITY primary study endpoint of safety through 30 days post-ablation procedure was previously published [13]. The report herein examines exploratory endpoints of local control and immunological changes, focusing on six-month radiographic outcomes and systemic immunophenotyping analyzed within the Per-Treatment Evaluable (PTE) population (Appendix B, Figure A1). Tumor response assessments were performed in 28 patients who underwent contrast-enhanced CT imaging at baseline (prior to ablation), and subsequently at 1-, 3- and 6-month visit windows after the ablation procedure in accordance with SOC institutional practices. Tumor response data is presented for the entire PTE population in Figure 2A and was stratified into two patient cohorts: patients who received no additional therapy following the index ablation procedure (open box patient ID in Figure 2A, *n* = 12 in Appendix B, Figure A1), and those who received systemic and/or focal therapies after the ablation index procedure (filled box patient ID in Figure 2A, *n* = 16 in Appendix B, Figure A1). Main baseline clinical characteristics for each patient cohort are presented in Appendix C (Table A1). No additional therapy was defined as therapy initiated after the index ablation procedure, including any antineoplastic or immunomodulating agents, radiation therapy, surgery, or a second ablation procedure targeting either a non-index lesion or a previously ablated tumor. Peripheral blood samples were available from 23 patients. Immunological analyses were limited to those who did not receive subsequent radiation or systemic therapy prior to sample collection, resulting in 13 patients included in the flow cytometry analysis and 11 patients in the IgG titer analysis (Appendix B, Figure A1). Three patients (A06, A23, A30) from the no additional therapy cohort described above were excluded from the immune biomarker analyses given that these patients received a somatostatin agonist (A06) or corticosteroids (A23 and A30) prior to sample collection, which could impact immune cell populations. Additionally, Supplementary Table S1 describe 4 patients (A14, A15, A16, A27) who were included in the immune biomarker analyses given that the subsequent therapy they received occurred after sample collection was completed. The Venn Diagram in Appendix B indicates the patients included in each of the analyses presented in this report. See Appendix A.1 for additional details.

### 2.4. Tumor Response Assessment

CT measurements were based on the most recent imaging available through the 6-month post-ablation visit window for each patient, using axial slices ≤ 5 mm to assess tumor burden as the sum of the longest diameters (SLD). Local response of each patient was evaluated with a modified version of Response Evaluation Criteria in Solid Tumor (RECIST) 1.1 [14,15] (mRECIST), where target lesions included only the ablated tumors and included those with longest diameters < 10 mm. Specific criteria for tissue-related characteristics such as cavitation or scar formation were not included in the evaluation reported here because they were not observed in the previously published clinical safety assessment from this study [13] or other prior studies [5]. Ablation coverage was defined as the ratio of the summed ablation volume to the baseline tumor volume, expressed as a percentage, as described in Appendix A.2. Non-ablated lesions were present in most patients (Table 1) and were not included in this initial local response evaluation. This focused approach was chosen to isolate the local effects of this form of ablation. Broader disease response, incorporating all measurable and non-target lesions, will be evaluated in the planned 12-month analysis.

### 2.5. Immune Biomarker Analyses

Immune biomarker analyses were conducted on the subset of patients that did not receive any additional antineoplastic, immunomodulating agents (including corticosteroids), or radiation therapy prior to blood collection for flow cytometry (up to ~30 days) or serological IgG evaluation (up to ~4 months). Biopsy samples were collected during the index ablation procedure. Blood and serum samples were collected at baseline (day 0), as well as at early (days 1–5), middle (days 6–16), late (days 17–43), and chronic (3 months) time points according to specifications in Supplementary Methods. Immunophenotyping of 29 circulating immune populations was performed on peripheral blood mononuclear cells (PBMCs) isolated from blood samples (Supplementary Methods, Supplementary Table S2). In addition, tumor biopsy-associated human IgG1 and tumor-associated antigens (TAAs) IgG circulating in patient sera were detected using an ELISA assay optimized in house (Supplementary Methods).

### 2.6. Statistical Analysis

Continuous variables are expressed as mean, standard deviation, number of patients, median, minimum, and maximum. Categorical variables are expressed as counts and percentages. Disease burden at baseline was compared between cohorts using a 2 × 3 contingency table and analyzed with Fisher’s exact test. Immunophenotyping data was analyzed using Wilcoxon matched pairs signed rank test on absolute values, which compares paired data between each patient time point relative to their baseline. Fold changes from baseline levels are reported as medians. IgG antibody levels were analyzed using unpaired t-tests with Welch’s correction, and statistical significance was determined based on q-values. Statistics were calculated using GraphPad Prism (RRID:SCR_002798, version 10.2.2, Boston, MA, USA).

## 3. Results

### 3.1. Procedural Results

From 31 enrolled patients, one was a screen failure prior to ablation as malignancy could not be confirmed intraprocedurally. One patient whose lesion was non-cancerous upon final pathology was excluded from analyses. Additionally, one patient died between the 3- and 6- month follow-up (cause unknown), with no radiographic or biospecimen data available. These exclusions resulted in a Per-Treatment Evaluable (PTE) population of 28 patients (Appendix B). Procedural success was achieved for all intended targets where navigation was successful in the PTE population. Details on the study’s safety endpoints have been previously reported, demonstrating an acceptable safety profile [13]. Briefly, the ablation procedure was well-tolerated, including in patients with bilateral lung lesions. Furthermore, this type of ablation near adjacent sensitive structures was feasible and safe. One procedure-related SAE (pneumothorax) resolved without sequelae, no AEs delayed first-line SOC therapy initiation, and CT scans at 30 days showed limited parenchymal changes around the ablation zones [13]. In summary, there was no additional risk from adding the ablation to the diagnostic procedure.

Table 1 reports baseline demographics, clinical characteristics, and ablation procedure details. Disease burden at baseline included solitary (Figure 1A), oligofocal, and multifocal (Figure 1B) presentations, defined as a single lesion; 2–5 lesions confined to one or two organs only; and six or more lesions or any number of lesions involving three or more organs, respectively. A total of 42 target lesions were ablated (range 1–3 lesions per patient). The mean number of activations per tumor was 3.4 ± 2.2 (range, 1–10). Histologies were grouped into colorectal, neuroendocrine, NSCLC, renal, sarcoma, and others. The most frequent histologies were colorectal and renal (*n* = 5 each), followed by NSCLC (*n* = 4). One patient presented with two histologies (NSCLC adenocarcinoma and neuroendocrine carcinoid).

### 3.2. 6-Month Follow-Up

At 6 months, two distinct patient cohorts emerged: those treated with ablation alone (*n* = 12) and those receiving additional systemic and/or focal therapies (*n* = 16) (Table 1, Figure 2, Table A1). Both groups included solitary, oligofocal, and multifocal cases and the distribution was similar between groups (*p* > 0.99) (Figure 2B, Table A1). Among the additional therapy group, 10 received systemic, 2 focal, and 4 both (Table 1). Additional therapy was defined as therapy initiated after the index ablation procedure, including any antineoplastic or immunomodulating agents, radiation therapy, surgery, or a second ablation procedure. Notably, ablation did not delay the initiation of other cancer treatments.

Figure 2A illustrates local control in ablated tumors at up to 6 months following energy delivery for both patient cohorts, along with the type and timing of systemic and/or focal therapies administered. The local response, clinical and tumor characteristics, and treatment details for the 12 patients who received ablation without any additional systemic or focal therapy are summarized in Table 2. Importantly, in these patients who did not receive additional treatment, ablation was associated with local control and appears to have delayed the need for systemic therapy. This cohort included the full spectrum of histologies, disease burden, number of tumors ablated, and procedural access types observed in the overall PTE population (Table 2A). Target tumor size ranged from 0.23 to 1.78 cm and all tumors received complete ablation coverage, requiring anywhere from 1 to 9 activations (Table 2B). The one exception was patient A26, whose tumor T1 (1.78 cm, right middle lobe, RML) received four activations for a 95% estimated ablation coverage, and tumor T2 (0.86 cm, right upper lobe, RUL) received two activations for a >100% estimated ablation coverage (see Appendix A.2, Table 2B). Despite both tumors achieving local control at the 3-month visit, the patient had a right middle lobectomy and upper lobe wedge resection a day after their 3-month follow-up visit. In addition to the two ablated tumors, the patient presented with a right lower lobe (RLL) groundglass nodule and an additional RUL cavitary nodule, which may have been a contributing factor in the decision to proceed with surgical intervention. At 6 months post-ablation, 33% (4/12) of patients without additional therapy achieved local partial response (PR), and 67% (8/12) achieved local stable disease (SD). In the additional therapy cohort, 1/16 (6%) reached local complete response (CR), 8/16 (50%) achieved local PR, and 6/16 (38%) achieved local SD. One patient (A13) had local disease progression (PD) and died of respiratory failure approximately 7.3 months post-ablation (Figure 2C). It is important to note that the study was not designed to randomize patients into these groups; rather, the two distinct cohorts emerged during data analysis. Both cohorts had comparable baseline clinical characteristics (Appendix C, Table A1), and no substantial differences in baseline disease burden were observed across ECOG performance status categories or vice versa (Appendix D, Table A2). However, given the non-randomized nature of the study, the observed results may reflect potential sources of bias in how patients were clinically managed following ablation. Long-term follow-up is ongoing, and 12-month RECIST outcomes for the same patients will be reported in a subsequent manuscript.

### 3.3. Immune Cell Dynamics

**Figure 3:**
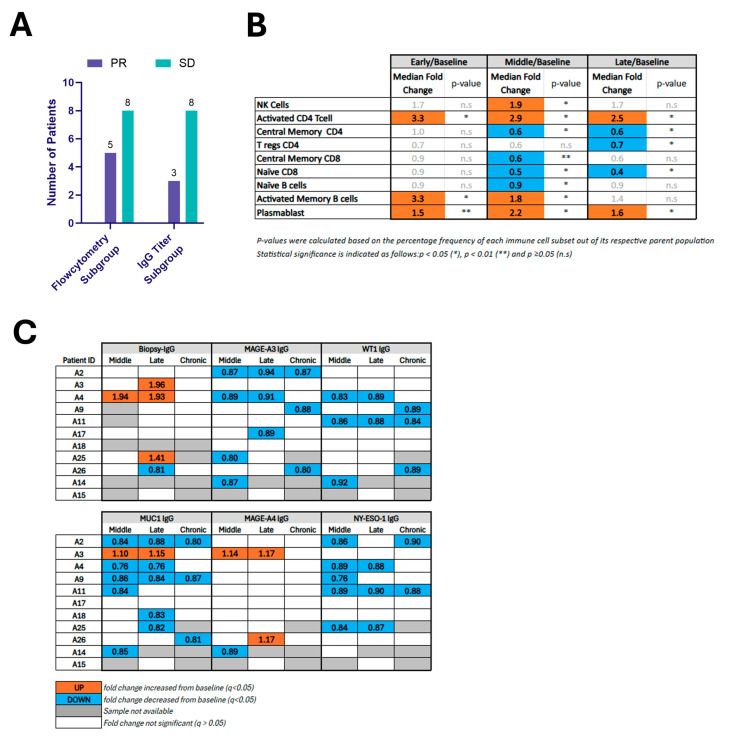
(**A**) Bar graph showing the number of patients with mRECIST-defined responses of ablated tumors among subgroups with available immune profiling data, including flow cytometry and IgG titer analysis. (**B**) Fold changes in systemic immune cell populations following ablation compared to baseline. This table reports median fold changes in immune populations at early, middle, and late time points relative to pre-ablation baseline in 13 patients. Only immune populations showing statistically significant changes at one or more time points are included. Populations were quantified by flow cytometry. (**C**) Changes in circulating IgG1 levels against biopsy-specific and tumor-associated antigens (TAAs) after ablation. IgG1 titers specific to the biopsy antigen and selected TAAs (MAGE-A3, WT1, MUC1, MAGE-A4, NY-ESO-1) were measured in patient serum at middle, late, and chronic time points post-ablation. Patients are labeled by ID (A2–A15). The table summarizes statistically significant differences from baseline.

All patients in the cohort analyzed by flow cytometry demonstrated local control by 6 months, with 5/13 achieving local PR and 8/13 achieving local SD (Figure 3A). Longitudinal changes were assessed in 29 immune cell populations. The profiling included major lymphocyte subsets (CD45+, NK-cells, NKT-cells, CD3+ T-cells, CD4+ T-cells, CD8+ T-cells), as well as subpopulations of CD4+ and CD8+ T-cells (effector memory, central memory, terminally differentiated effector memory, naïve, and exhausted PD-1+ cells). The gating strategy used to identify T-cell subsets is shown in Supplementary Figure S1. Regulatory T-cells (CD4+ T_regs_ (CD4+ CD25+ CD127-)) were analyzed within the CD4+ population. B-cell subsets (naïve, memory, activated memory, plasma cells, and plasmablasts), and immunoglobulin-producing B-cell populations (switched, unswitched, IgG+ B-cell, IgM+ B-cell) were characterized using the gating strategy illustrated in Supplementary Figure S2.

Figure 3B reports the median fold change and *p*-value for the 9 immunocyte populations that demonstrated statistically significant changes relative to baseline for at least one time point. NK-cells increased during the middle phase, activated CD4^+^ CD28^+^ T-cells were significantly elevated at all time points (early, middle, and late). Activated Memory B-cells showed significant increases at both early and middle phases, and plasmablasts were elevated across all three time points. In contrast, decreases were found in T_regs_ at the late phase, naïve CD8^+^ T-cells (during the middle and late phases), naïve B-cells in the middle phase and Central Memory CD4^+^ T-cells at both the middle and late time points. The scatter plot in Supplementary Figure S3 depicts each patient’s absolute value (Supplementary Figure S3A) as well as median fold-change values (Supplementary Figure S3B) for the cell populations that reached statistical significance versus baseline. A further 20 immunocyte populations among the 29 analyzed did not show significant changes over time compared to baseline (Supplementary Table S3).

### 3.4. Biopsy-Specific IgG and TAA-Specific Antibody Profiling

Of the 12 patients without additional therapy, only 9 had matched serum and biopsy samples to assess biopsy-specific IgG. Of the 12 patients in this cohort, only11 had baseline and follow-up serum available for assessing changes in tumor-associated antigen (TAA)-specific IgG. All patients in this cohort demonstrated local control by 6 months, with 3/11 achieving local PR and 8/11 achieving local SD (Figure 3A).

For biopsy-specific antibodies, 33% (3/9) exhibited a ≥20% rise in biopsy-specific IgG1 at ≥1 post-treatment time point (Figure 3C and Supplementary Table S4), with maximal activity within the first month following intervention, and one subject showing sustained increase in two consecutive time points (middle and late). No patients exhibited statistically significant modulation of IgG levels at the chronic time point, although one patient with a late time point rise had no chronic time point sample available for analysis. The magnitude of response varied between 41% and 96% increase over baseline. One patient had a 19% decrease in biopsy-specific IgG at the late time point.

Figure 3C reports the direction of statistically significant changes in selected TAA-specific IgG levels for each patient following ablation (see also Supplementary Table S4). Reduced MAGE-A3-, WT1-, and NY-ESO-1-specific IgG was observed in 7, 5, and 5 patients, respectively. MAGE-A4-specific IgG titer was modulated in three patients (2 increased, 1 decreased). MUC1-specific IgG titer was modulated in nine patients (1 increased, 8 decreased).

## 4. Discussion

The AFFINITY trial demonstrated that ablation of lung lesions at the time of diagnostic biopsy was safe, with only one pneumothorax requiring chest tube from a percutaneous case and, importantly, did not delay institutional standard of care subsequent therapy [13]. Here, we report details on medium-term (6-month) local control rates and immunological changes in patients from the trial.

Local control rates of lesions following ablation were 12/12 (100%) for patients who did not receive additional therapy and 15/16 (94%) for those who did. These high response rates in both cohorts suggest that Aliya PEF ablation may provide effective local control of lesions ablated at the time of diagnosis, including bilateral lung lesions in the same setting, though significant longer follow-up and larger studies are needed.

Notably, despite the diverse histologic subtypes and varying disease burden, local control rates of stability or partial response demonstrate the broad applicability of this ablation and continue to build upon its evidence of consistent ablation zone regardless of location within the lung [13]. Further, 12 patients did not receive subsequent additional therapy by the 6-month follow-up imaging. This is significant given the advanced, Stage IV disease in the patients included in the trial. For example, stage IV NSCLC patients may have a median survival of as little as 4 months without treatment [16,17]. Thus, while these patients would normally undergo subsequent therapy, particularly in cases of oligofocal and multifocal disease (*n* = 11/12), the clinicians determined no additional therapy was warranted at the time. All but one targeted lesion from these patients were completely ablated; the remaining lesion had an estimated ablation coverage of 95%, highlighting the importance of comprehensive tumor coverage to achieve local tumor control. Cancer progression is frequently observed at the primary site of disease in oligometastatic stage IV lung cancer patients, highlighting the importance of local control in this setting [16,17]. Given that 10 patients from this group had additional tumors that did not receive ablation nor subsequent therapy, it suggests there might be a systemic impact of this specialized energy.

In addition to evaluating local control, this study characterizes the temporal dynamics of systemic immune responses following ablation, specifically for the patients who did not undergo subsequent therapy. Key adaptive immune populations were assessed via flow cytometry, with changes evaluated relative to baseline. In the early phase (3–5 days post-ablation), immune activation likely reflected responses to novel and known tumor antigens, with reactivated memory B-cell and clonal expansion of activated CD4^+^ T-cells. In the middle phase (6–16 days post-ablation), eight populations shifted, including reductions in central memory and naïve CD8^+^ T-cells, suggesting tissue migration and activation. In the late phase (17–43 days post-ablation), these trends persisted, with sustained increases in plasmablasts/plasma cells, reduced Tregs, and elevated activated CD4^+^ T-cells. Overall, this profile suggests a robust activation of both adaptive and innate immunity. The observed expansion of memory/effector T-cells indicates a shift toward active immune engagement [18], aligning with preclinical murine data showing increases in circulating CD8^+^ T-, B-, and NK-cells [6,9,10]. Extended follow-up is needed to determine the durability of these responses, but this pattern is consistent with immunogenic therapies like cancer immunotherapy or vaccination [19,20].

Serum IgG1 profiling using biopsy lysate ELISA showed that 33% of ablation-only patients mounted a detectable humoral response. Analysis of TAA-specific IgG titers revealed distinct patterns of modulation: 18% of patients showed an increase in MAGE-A4-specific IgG, while 45% demonstrated a decrease in WT1 IgG, 45% in NY-ESO-1 IgG, and 72% in MUC1 IgG. These antigens, typically silent in healthy tissues, can elicit spontaneous adaptive responses [21,22,23,24]. Serological detection of IgG against these tumor-associated antigens has been previously reported in the literature, with associations to both favorable and unfavorable clinical outcomes depending on the context. IgG increases against MUC1 and MAGE-A4 were reported in other studies and have been linked to favorable outcomes in other cancers, including NSCLC [25,26]. Although, an increase in antigen-specific IgG does not necessarily equate to functional antibody-mediated recognition or clearance of tumor cells. Many tumor-associated antigens, such as cancer-testis antigens and intracellular oncoproteins, are not expressed on the surface of intact tumor cells, rendering them inaccessible to circulating antibodies [27,28]. Consequently, although antibodies may bind to their target antigens in lysates or after cell death, they are unlikely to mediate direct cytotoxic effects unless the antigen is displayed on the cell membrane. Thus, while post-ablation increases in antigen-specific IgG could reflect a boosted humoral immune response, they should be interpreted primarily as biomarkers of immune engagement rather than direct effectors of antitumor activity. 

These analyses have limitations. The protein lysate derived from biopsy tissue contains a mix of tumor cells, healthy cells, and exogenous proteins, introducing background noise; and only IgG1 was measured, excluding other relevant subclasses [29]. The lack of a control group limits interpretation of immune fluctuations, such as baseline variability, temporal changes in IgG levels, or shifts in immune cell composition, which may occur independently of ablation.

Another limitation is the relatively short (6-month) follow-up period following ablation which makes it challenging to determine potential relationships between the overall radiologic outcomes and immunological changes. Tumor progression, recurrence, the onset of new lesions, or observation of responses in non-ablated lesions may not yet be present. In addition, there is a broad range of underlying cancers present within the study population, where each may have unique downstream immunological implications. Moreover, only radiographic responses of ablated targets are reported here, providing indications of local control which may occur independently from immunological effects. Lastly, as the study was non-randomized and the two cohorts emerged during analysis, the results may reflect potential sources of bias in post-ablation patient management. While some of these limitations are inherent to the study design, the 12-month analysis is warranted to evaluate potential associations once longer-term definitive response and survival data are available.

## 5. Conclusions

In summary, this study provides compelling evidence that this form of ablation is able to attain local control in ablated tumors in the setting of stage IV disease and may additionally induce a tumor-specific immune response, as evidenced by perturbation of biopsy-specific antibody production and favorable adaptive immune cell dynamics. Unlike thermal ablation, this form of ablation does not cause coagulation of structural and cellular proteins as a result of extreme temperatures, potentially allowing for enhanced immune recognition and response [30]. The data provided here suggest that this specialized energy activates humoral immunity, leading to increased production of biopsy-specific antibodies, as well as modulation of general TAA-IgG in some patients. This immune modulation may have significant implications for integrating focal ablation with immunotherapy strategies, such as checkpoint inhibition. By priming the immune system through this specialized energy, concurrent or subsequent immunotherapy may yield enhanced clinical benefits. These findings suggest that this form of ablation could play a crucial role in modulating the cancer immunity cycle and enhancing systemic oncologic outcomes beyond local tumor control. Further research is warranted to validate these findings and explore the integration of this form of ablation with emerging immunotherapeutic strategies.

## Figures and Tables

**Table 1:** Baseline Demographics, Clinical, and Procedural Characteristics (PTE Population) *n* = 28 Patients.

Median Age at Consent [range]	66 [28, 85]
Sex (% patients) Female Male	14 (50.0%) 14 (50.0%)
BMI (mean ± SD)	26.7 ± 5.6
ECOG Performance Status (% patients) 0 1 2 3 Not Available	20 (71.4%) 6 (21.4%) 0 (0.0%) 1 (3.6%) 1 (3.6%)
Malignancy Group (% patients) Colorectal Neuroendocrine NSCLC Other ^1^ Renal Sarcoma	5 (17.9%) 3 (10.7%) 4 (14.3%) 9 (32.1%) 5 (17.9%) 2 (7.1%)
Disease Burden at Baseline (% patients) Solitary Oligofocal Multifocal	2 (7.1%) 8 (28.6%) 18 (64.3%)
Total Lung Tumors Ablated with PEF	42
Mean Tumor Size-Longest Diameter ± SD [range] (cm)	1.1 ± 0.5 [0.4, 2.4]
Mean Number of PEF Activations per Tumor ± SD [range]	3.4 ± 2.2 [1, 10]
Number of Tumors Ablated in the Same Session (% patients) 1 2 3	17 (60.7%) 8 (28.6%) 3 (10.7%)
Additional Treatment post-PEF before 6mo Imaging (% patients) No Focal or Systemic Treatment Focal Treatment Only Systemic Treatment Only Both Focal and Systemic Treatment	12 (42.9%) 2 (7.1%) 10 (35.7%) 4 (14.3%)

PTE = per-treatment evaluable; PEF = pulsed electric fields; BMI = body mass index; SD = standard deviation; ^1^ Patient A26 is reported as ‘Other’ given presentation with two histologies: NSCLC adenocarcinoma and Neuroendocrine carcinoid.

**Table 2:** Clinical and tumor characteristics and treatment response of ablation-only patients. (**A**): Clinical Characteristics and Treatment Response of PEF-Only Patients (*n* = 12 patients). (**B**): Tumor Characteristics and Treatment of PEF-Only Patients (*n* = 12 Patients).

**(A)**						
**Patient ID/Sex/Age (yrs)**	**Malignancy Designation**	**Disease Burden at Baseline**	**Total Tumors Identified in the Lung**	**Number of PEF Ablated Tumors**	**PEF Energy Delivery Approach**	**mRECIST Patient Response at Last Imaging up to 6 months**
A25/77/F	Adenocarcinoma, unknown primary	Solitary	1	1	Bronchoscopic	PR
A23/65/F	Neuroendocrine carcinoid	Oligofocal	2	1	Bronchoscopic	SD
A09/82/M	Oropharyngeal squamous cell carcinoma	Oligofocal	2	2	Bronchoscopic	SD
A03/77/F	Renal cell carcinoma	Oligofocal	2	1	Bronchoscopic	SD
A18/74/M	Renal cell carcinoma	Multifocal	2	1	Bronchoscopic	PR
A06/75/F	Neuroendocrine carcinoid	Multifocal	6	1	Bronchoscopic	PR
A04/76/F	Neuroendocrine carcinoid	Multifocal	4	1	Bronchoscopic	PR
A26/76/F	NSCLC adenocarcinoma; Neuroendocrine carcinoid	Multifocal	>5	2	Bronchoscopic	SD
A11/85/M	Papillary thyroid carcinoma	Multifocal	>5	3	Bronchoscopic	SD
A02/66/M	Papillary thyroid carcinoma	Multifocal	5	1	Bronchoscopic	SD
A17/64/M	Renal cell carcinoma	Multifocal	>5	2	Percutaneous	SD
A30/85/M	Renal cell carcinoma	Multifocal	2	2	Bronchoscopic	SD
**(B)**						
**Patient ID**	**Tumor ID**	**Tumor Location**	**Tumor Longest Diameter (cm) at Baseline**	**Number of PEF Activations**	**Ablation Coverage (%)**	**mRECIST Patient Response at Last Imaging up to 6 months**
A25	A25_T1	RUL	0.99	3	>100%	PR
A23	A23 T1	LUL	1.00	2	>100%	SD
A09	A09_T1	LUL	1.77	6	>100%	SD
	A09_T2	RUL	0.88	1	>100%	SD
A03	A03_T1	RLL	1.31	8	>100%	SD
A18	A18_T1	RLL	1.72	3	>100%	PR
A06	A06_T1	RML	1.23	5	>100%	PR
A04	A04_T1	RML	1.36	7	>100%	PR
A26	A26_T1	RML	1.78	4	<100%	SD
	A26_T2	RUL	0.86	2	>100%	PR
A11	A11_T1	LLL	1.13	4	>100%	SD
	A11_T2	RML	1.10	3	>100%	SD
	A11_T3	RUL	1.12	2	>100%	SD
A02	A02_T1	LUL	1.57	9	>100%	SD
A17	A17_T1	RLL	0.93	3	>100%	SD
	A17_T2	RLL	0.23	1	>100%	SD
A30	A30_T1	LUL	0.86	2	>100%	SD
	A30_T2	RUL	0.41	1	>100%	SD

PEF = pulsed electric fields; F = female; M = male; mRECIST = modified Response Evaluation Criteria in Solid Tumors; SD = stable disease; PR = partial response; solitary = single lesion identified; oligofocal = 2–5 lesions confined to one or two organs only; multifocal = six or more lesions or any number of lesions involving three or more organs. RUL = right upper lobe; RML = right middle lobe; RLL = right lower lobe; LUL = left upper lobe; and LLL = left lower lobe.

## Data Availability

Written requests with methodologically reasonable proposals for access to study data will be reviewed and may require data use agreements. Any data provided will be de-identified, and in compliance with applicable privacy laws as well as data protection, consent, and anonymization requirements. Requests should be directed to Galvanize Therapeutics.

## Supplementary Materials

The following supporting information can be downloaded at: https://www.mdpi.com/article/10.3390/cancers17213495/s1; Figure S1: Gating strategy for identification of T-cell subpopulations; Figure S2: Gating strategy for identification of B-cell subpopulations; Figure S3: Individual patient immune cell responses over time points with significant changes from baseline; Table S1: Clinical and tumor characteristics and treatment response of patients with focal or systemic therapy delivered after blood collection for flow cytometry and IgG titer; Table S2: List of antibodies used for flow cytometry analysis of B- and T-cell subpopulations; Table S3: The table reports immune populations profiled by flow cytometry that did not show statistically significant differences compared to baseline; Table S4: Longitudinal profiling of circulating IgG1 responses in individual patients.

## Abbreviations

AE	Adverse Events
BMI	Body Mass Index
BSA	Bovine Serum Albumin
CR	Complete Response
CT	Computed Tomography
DAMP	Damage-Associated Molecular Pattern
DMSO	Dimethyl Sulfoxide
ELISA	Enzyme-Linked Immunosorbent Assay
FIH	First in Human
IgG	Immunoglobulin G
LLL	Left Lower Lobe (lung anatomy)
LUL	Left Upper Lobe (lung anatomy)
MRI	Magnetic Resonance Imaging
mRECIST	Modified Response Evaluation Criteria in Solid Tumor
NSCLC	Non-Small Cell Lung Cancer
OD	Optical Density
PBMC	Peripheral Blood Mononuclear Cells
PBS	Phosphate-Buffered Saline
PEF	Pulsed Electric Field
PET-CT	Positron Emission Tomography-Computed Tomography
PD	Progressive Disease
PR	Partial Response
PTE population	Per-Treatment Evaluable population
RLL	Right Lower Lobe (lung anatomy)
RML	Right Medial Lobe (lung anatomy)
RUL	Right Upper Lobe (lung anatomy)

## Appendix A

### Appendix A.1. Additional Analysis Groups Details

Additional therapy excluded systemic therapies not impacting cancer status and ongoing at enrollment. Three patients presented with unresolved disease activity, requiring diagnostic biopsy and were receiving systemic therapy at enrollment: A06 (lanreotide), A26 (anastrozole) and A20 (pembrolizumab) and are indicated by * in Figure 2A. The cohort assignment was based on measurements from the last available and evaluable CT scan through the 6-month visit window. Patient A26 underwent surgery one day after their 3-month imaging, considered their last evaluable CT scan, and thus was assigned to the no additional therapy cohort. Post-surgery imaging available from this patient was considered non-evaluable and was excluded from mRECIST response analysis. If the start of additional therapy occurred after the index ablation procedure and prior to any evaluable CT scan for that patient, the patient was assigned to the additional therapy cohort. The type and timing of the additional therapy administered is also presented in Figure 2A, with data available through the 6-month visit window for each patient. For clarification, patient A23 initiated targeted therapy 5.6 months after ablation, which is within the 6-month visit window, but the last available and evaluable CT scan was from the 3-month visit, so the patient was stratified into the no additional therapy cohort.

### Appendix A.2. Ablation Coverage Estimation

Ablation coverage was calculated per tumor, as a percentage of the total ablation volume relative to the tumor volume at baseline, modeling both the tumor and ablation zone as ellipsoids. Tumor volume was estimated using the two axial measurements used for mRECIST, with the third dimension approximated as the average of the two available measurements. Ablation zone dimensions were based on the manufacturer’s Instructions for Use with the third dimension similarly estimated as the average of the two cross-sectional measurements.

Total ablation volume for each tumor was calculated by multiplying the volume of a single ablation zone by the number of activations delivered, incorporating an assumed 25% overlap between ablation zones. This overlap adjustment accounts for the fact that ablation zones may intersect, reducing the effective ablation volume. Because the true extent of overlap is variable and not directly measurable, the 25% assumption was used to represent a conservative estimate of tumor coverage, as opposed to assuming no overlap. Ablation zones extended beyond tumor boundaries, and coverage percentages were not capped at 100%.

## Appendix C

**Table A1:** Main baseline clinical characteristics between No Additional Therapy (*n* = 12) and Additional Therapy (*n* = 16) patient cohorts. Percentages represent the proportion of patients within each therapy group who met the baseline characteristics.

	No Additional Therapy Patients *n* (% Patients)	Additional Therapy Patients *n* (% Patients)
**ECOG**		
0	8 (66.7%)	12 (75.0%)
1	3 (25.0%)	3 (18.8%)
3	0 (0.0%)	1 (6.3%)
Not Available	1 (8.3%)	0 (0.0%)
**Malignancy Group**		
Colorectal	0 (0.0%)	5 (31.3%)
Neuroendocrine	3 (25.0%)	0 (0.0%)
NSCLC	0 (0.0%)	4 (25.0%)
Other	5 (41.7%)	4 (25.0%)
Renal	4 (33.3%)	1 (6.3%)
Sarcoma	0 (0.0%)	2 (12.5%)
**Disease Burden at Baseline**		
Solitary	1 (8.3%)	1 (6.3%)
Oligofocal	3 (25.0%)	5 (31.3%)
Multifocal	8 (66.7%)	10 (62.5%)
**Number of Tumors Ablated**		
1	7 (58.3%)	10 (62.5%)
2	4 (33.3%)	4 (25.0%)
3	1 (8.3%)	2 (12.5%)

## Appendix D

**Table A2:** Disease burden at baseline based on ECOG between No Additional therapy (*n* = 12) and Additional Therapy (*n* = 16) patient cohorts. Percentages represent the proportion of patients within each therapy group who met the combined baseline characteristics.

	No Additional Therapy Patients *n* (% Patients)	Additional Therapy Patients *n* (% Patients)
**ECOG = 0**		
Solitary	1 (8.3%)	1 (6.3%)
Oligofocal	2 (16.7%)	4 (25.0%)
Multifocal	5 (41.7%)	7 (43.8%)
**ECOG = 1**		
Oligofocal	1 (8.3%)	0 (0.0%)
Multifocal	2 (16.7%)	3 (18.8%)
**ECOG = 3**		
Oligofocal	0 (0.0%)	1 (6.3%)
**ECOG—Not available**		
Multifocal	1 (8.3%)	0 (0.0%)
