# Supplementary material for: Six-Month Local Control Rates and Immune Responses After Pulsed Electric Field Ablation in Metastatic Cancer
Source: Cancers (Basel). 2025 Oct 30;17(21):3495. doi: 10.3390/cancers17213495 (PMC12608876; doi:10.3390/cancers17213495)
Supplement: Supplementary file 1 [file cancers-17-03495-s001.zip › cancers-3917319-supplementary_2nd proof.pdf]

cancers

MDPI

Article

Six-Month Local Control Rates and Immune Responses After Pulsed Electric Field Ablation in Metastatic Cancer

Alicia Moreno-Gonzalez [1], Ebtesam H. O. Nafie [1], Chiara Pastori [1], Joseph Mammarappallil [2], Partha Seshaiah [1], Maria B. Plentl [1], Beryl A. Hatton [1], Robert E. Neal II [1], Michael A. Pritchett [3], Janani S. Reisenauer [4], Sebastian Fernandez-Bussy [5], David DiBardino [6], Bradley B. Pua [7] and William S. Krimsky [1,*]

Supplementary Materials:

PBMCs Isolation

Peripheral blood mononuclear cells (PBMCs) were isolated from freshly collected EDTA-anticoagulated blood (2x10 ml tubes) by density gradient centrifugation. In brief, whole blood collected in anticoagulant-coated tubes was diluted 1:1 with phosphate-buffered saline (PBS) and carefully poured into 50 ml SepMate™-50 tubes (StemCell Technologies, cat. 15420) pre-filled with 15 ml Lymphoprep™ (cat. 07801). Samples were centrifuged at 400 × g for 30 min at room temperature with the brake set to level 6 (acceleration off), the distinct PBMC layer at the plasma Lymphoprep interface was aspirated and transferred to new tubes then washed twice with PBS (300 × g, 10 min) to remove residual plasma and platelets. After the final wash, cells were resuspended in complete RPMI-1640, counted by trypan-blue. For cryopreservation, PBMCs were mixed with 1ml chilled freezing medium (90% fetal bovine serum + 10% dimethyl-sulfoxide [DMSO]), gently vortexed, and dispensed into cryovials. Vials were placed in a controlled-rate freezing container (−1°C min^{-1}) at −80°C overnight, then transferred to the liquid phase of liquid nitrogen for long-term storage and subsequent batch analyses.

Immunophenotyping of PBMCs by Flow Cytometry

Cryopreserved PBMC aliquots (~2 × 10^6 cells) were thawed in a 37°C water bath, washed twice in wash buffer (PBS + 1% bovine serum albumin (BSA)), and resuspended in 100 µl of BioLegend Cell Staining Buffer (cat. 420201). Cells were incubated on ice for 30 min in the dark with a pre-titrated antibody cocktail and Zombie NIR viability dye (BioLegend, cat. 423105); antibody clones, fluorochromes, and catalogue numbers are listed in Supplementary Table S2. After staining, cells were washed twice in the same PBS/BSA wash buffer (400 × g, 5 min, 4°C) and fixed for 15 min in 1% paraformaldehyde/PBS at room temperature. Samples were acquired on a CytoFLEX flow cytometer (Beckman Coulter) equipped with three lasers (405, 488, and 638 nm). A single-stain-bead compensation matrix was generated once and applied to all files. PBMCs from every patient's baseline (Day 0) sample were included in each instrument run to minimize inter-assay variability across batched analyses. A minimum of 5 × 10^5 live $CD45^+$ events were recorded at low -medium flow rate. Debris and doublets were excluded by FSC/SSC and FSC-H vs FSC-A gating; dead cells were removed by Zombie NIR uptake (Supplementary Figure S1-S2). Subsequent hierarchical gates resolved: T-cell panel (Supplementary Figure S1) – $CD3^+$ → $CD4^+$/$CD8^+$ subsets; naïve, central-memory, effector-memory, TEMRA, regulatory ($CD4^+$CD25^hiCD127^lo), activated ($CD28^+$) and PD-1^+ fractions; NK ($CD3^-CD56^+$) and NKT-cells ($CD3^+CD56^+$). B-cell panel (Supplementary Figure S2) – $CD19^+$ → naïve (IgD^+CD27^-), unswitched memory (IgD^+CD27^+), switched memory

 https://doi.org/10.3390/cancers17213495

(IgD⁻CD27⁺), double-negative memory (IgD⁻CD27⁻) and plasmablasts/plasma cells (CD20^lo/⁻CD38^hiCD138⁺); and isotype-defined (IgG/IgM) memory compartments. All gating and statistics were performed with CytExpert 3.x. Frequencies for each subset were exported for downstream statistical analyses.

Serum Isolation

Peripheral blood (~6 mL each) was drawn into red-top Vacutainer® tubes (BD, Cat. 367815) containing no anticoagulant. The tubes were kept upright at room temperature for approximately 20–30 minutes to allow complete clot formation, then centrifuged according to the site's SOP (typically 1,300 × g, 10 min, 20°C). The clear serum fraction was carefully aspirated, transferred to sterile, prelabeled cryovials, and immediately frozen at –80°C for long-term storage pending batch analysis.

Extraction of total protein from tumor biopsies

Tumor samples were collected during the biopsy procedure performed under image guidance prior to energy delivery. The specimens obtained were immediately snap-frozen and stored at -80°C for subsequent analysis. Total protein was extracted from biopsy specimens using cell lysis buffer (RIPA buffer) to extract and solubilize proteins. Briefly, tissue samples were mechanically digested using a tissue homogenizer in a microcentrifuge tube containing lysis buffer supplemented with a protease and phosphatase inhibitor cocktail to prevent protein degradation and dephosphorylation. Samples were further homogenized on ice using a mechanical homogenizer and then were incubated on ice for 30 minutes to enhance protein solubilization. The homogenates were then centrifuged at 12,000 × g for 15 minutes at 4°C to pellet cellular debris, and the supernatants containing the total protein extracts were carefully collected and stored at −80°C until further analysis.

Detecting Immunoglobulin G (IgG) by ELISA

One 6 ml serum tube was collected and processed by the site laboratory to obtain serum. Human IgG1 circulating in patient sera were detected using an ELISA assay optimized in house. Briefly, 96-well microtiter plates were coated in triplicate overnight at 4°C with 100μL of proteins extracted from each patient's biopsy specimen. The following day, the protein lysate was removed from each well and after three washes with PBS, the wells were subjected to a blocking step with 1% BSA in PBS to prevent non-specific binding. Serum samples were diluted 1:50 in PBS, added in triplicate and incubated at room temperature for 2 hours. Next, after removing the sera, the wells are washed extensively with PBS containing 0.05% Tween-20 and an HRP-conjugated anti human-IgG1 detection antibody (Jackson ImmunoResearch Laboratories, 109-035-088) was applied and incubated for 1 hour. Plates were then washed and incubated with tetramethylbenzidine (TMB) substrate for 15 minutes and the reaction was stopped with STOP buffer. Absorbance was measured at 450 nm using a microplate reader and the IgG1 levels were quantified by optical density (O.D.) calculations (or interpolation from a standard curve). ELISA plates were also coated with synthetic peptides corresponding to the following selected tumor-associated antigens (TAAs) for the detection of antigen-specific IgG in patient serum samples: NY-ESO-1 (BPS Bioscience, 78758), MAGE-A3 (Miltenyi, 130-095-384), MAGE-A4 (BPS Bioscience, 78966), WT1 (JPT, PM-WT1), MUC1 (Abcam, ab94833). The lyophilized tumor-associated antigens (TAA) were resuspended in PBS at the concentration of 1 mg/ml and diluted in PBS (1:1000) to be plated in 96-well plate (100 μL/well) to perform detection of TAA-specific IgG by ELISA as described above. IgG1 was quantified in serum at baseline and at three post-intervention time-points: middle, late and chronic. O.D. values were normalized to each patient's baseline reading; fold-change ≠ 1 combined with a paired-sample t-test ($p < 0.05$) was considered significant.

Supplementary Tables and Figure:

Supplementary Table S1. Clinical and tumor characteristics and treatment response of patients with focal or systemic therapy delivered after blood collection for flow cytometry and IgG titer.

Supplementary Table S1A: Clinical Characteristics and Treatment Response of Patients with Focal or Systemic Therapy Delivered After Blood Collection for Flow Cytometry and IgG Titer (*N=4 patients)*

Patient ID/Sex /Age (yrs)	Malignancy Designation	Disease Burden at Baseline	Total Tumors Identified in the Lung	Number of PEF Ablated Tumors	PEF Energy Delivery Approach	mRECIST Patient Response at Last Imaging up to 6mo	Additional Therapy After Sample Collection for Flow Cytometry and IgG Titer
**A15**/45 /M	Colorectal adenocarcinoma	Multifocal	>5	2	Bronchoscopic	SD	Radiation of pre-existing non-PEF tumor, targeted therapy, chemotherapy
**A16**/79 /M	NSCLC adenocarcinoma	Multifocal	5	1	Bronchoscopic	PR	Targeted therapy
**A27**/66 /M	NSCLC squamous cell carcinoma	Multifocal	>5	2	Bronchoscopic	PR	Radiation of previously PEF ablated tumor and pre-existing non-PEF tumor
**A14**/32 /F	Synovial sarcoma	Multifocal	>5	3	Bronchoscopic	SD	Chemotherapy

PEF = pulsed electric fields; F = female; M = male; mRECIST = modified Response Evaluation Criteria in Solid Tumors; SD = stable disease; PR = partial response; solitary = single lesion identified; oligofocal = 2-5 lesions confined to one or two organs only; multifocal = six or more lesions or any number of lesions involving three or more organs

Supplementary Table S1B: Tumor Characteristics and Treatment of Patients with Focal or Systemic Therapy Delivered After Blood Collection for Flow Cytometry and IgG Titer (*N=4 Patients)*

Patient ID	Tumor ID	Tumor Location	Tumor Longest Diameter (cm) at Baseline	Number of PEF Activations	Ablation Coverage (%)	mRECIST Patient Response at Last Imaging up to 6mo	Additional Therapy After Sample Collection for Flow Cytometry and IgG Titer
**A15**	A15_T1	RUL	1.33	5	>100%	SD	Targeted therapy, chemotherapy
	A15_T2	LUL	2.66	6	>100%	PR	Targeted therapy, chemotherapy
**A16**	A16_T1	RLL	1.30	4	>100%	PR	Targeted therapy
**A27**	A27_T1	RLL	0.57	1	>100%	CR	Targeted therapy
	A27_T2	LLL	1.22	4	>100%	PR	Radiation of previously PEF ablated tumor
**A14**	A14_T1	RLL	1.22	3	>100%	SD	Chemotherapy
	A14_T2	RML	0.82	2	>100%	SD	Chemotherapy
	A14_T3	LLL	1.15	2	>100%	SD	Chemotherapy

PEF = pulsed electric fields; mRECIST = modified Response Evaluation Criteria in Solid Tumors; SD = stable disease; PR = partial response; RUL = right upper lobe; RML = right middle lobe; RLL = right lower lobe; LUL = left upper lobe; and LLL = left lower lobe.

Supplementary Table S2. List of antibodies used for flow cytometry analysis of B- and T-cell subpopulations.

Specificity/Ab	Fluorochrome	Clone	Vendor	Catalog	Purpose
**T cell Panel**					
Zombie NIR	APC-CY7	-	Biolegend	423105	Viability
CD45	BV510	HI30	Biolegend	304036	Leukocytes
CD3	APC	OKT3	Biolegend	317318	T cell, NKT-Like cells
CD4	PE	RPA-T4	Biolegend	300508	CD4 T cells
CD8	PB-450	SK1	Biolegend	344718	CD8 T cells
CD56	PerCP5.5	5.1H11	Biolegend	362506	NK cells
CD25	Alexa fluor700	M-A251	BD Bioscience	561398	Regulatory T cells
CD127	PE-CY7	A019D5	Biolegend	986008	T cell differentiation
CD45RA	FITC	HI100	Biolegend	304106	T cell differentiation
CD27	PE-Dazzle594	LG.3A10	Biolegend	124228	T cell differentiation
CCR7	BV605	2-L1-A	BD Bioscience	566755	T cell differentiation
PD-1	BV650	EH12.1	BD Bioscience	564104	T cell inhibitory receptor
CD28	BV785	CD28.2	Biolegend	302950	T cell differentiation
**B cell panel**					
CD19	APC	HIB19	Biolegend	302212	B cells
CD20	PB-450	2H7	Biolegend	302328	B cells
IgG	FITC	CBRM1/5	Biolegend	301404	B cell differentiation
IgM	BV650	MHM-88	Biolegend	314526	B cell differentiation
IgD	BV605	IA6-2	Biolegend	348232	B cell differentiation
CD3	PE	UCHT1	Biolegend	300408	T cells
CD14	PE	M5E2	Biolegend	301806	Monocyte differentiation
CD56	PE	5.1H11	Biolegend	362507	NK cells
CD27	PE-Dazzle594	LG.3A10	Biolegend	124228	B cell differentiation
CD21	PerCP5.5	Bu32	Biolegend	354908	B cell differentiation

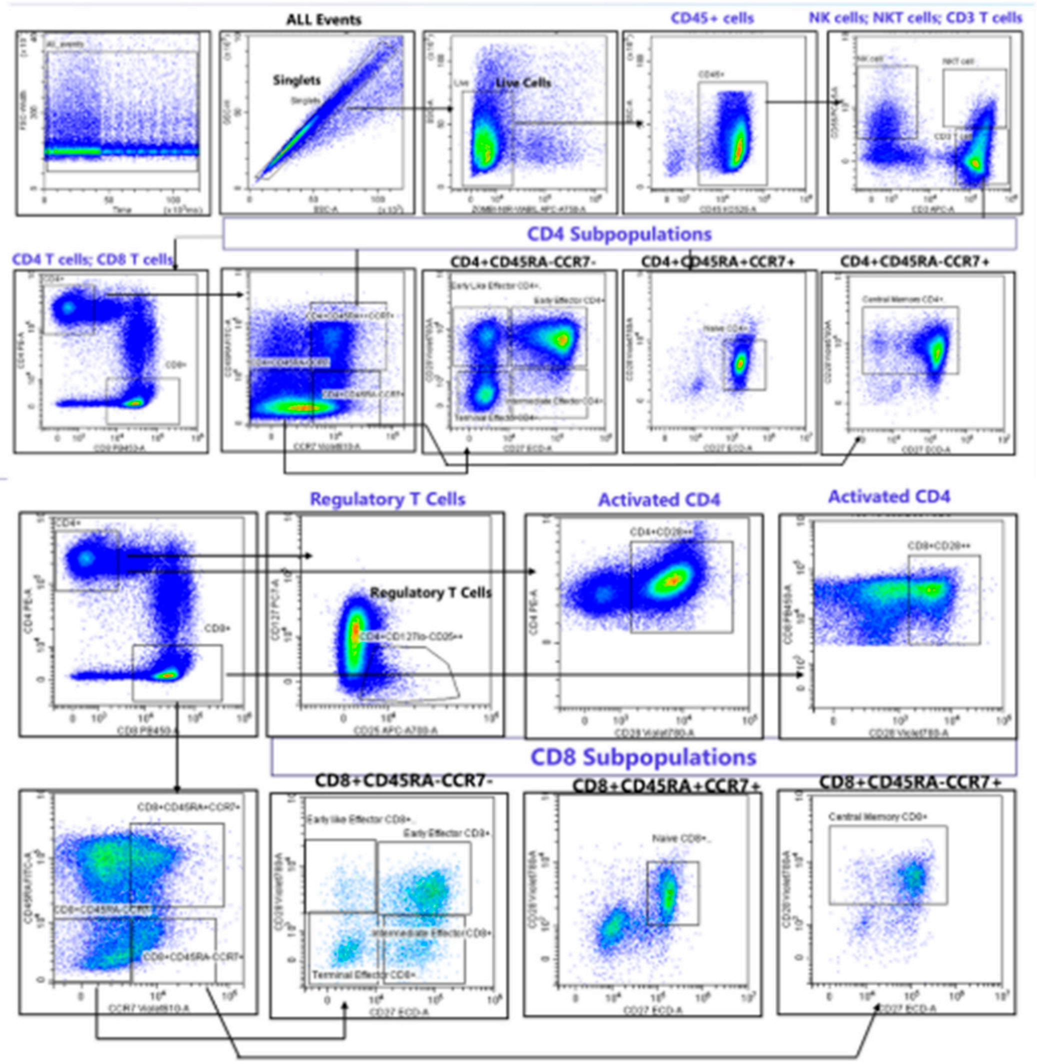

Supplementary Figure S1. Gating strategy for identification of T-cell subpopulations. Flow cytometry gating was performed on live, singlet, $CD45^+$ leukocytes. $CD3^+$ T-cells, NK-cells ($CD3^-CD56^+$), and NKT-cells ($CD3^+CD56^+$) were identified. $CD3^+$ T-cells were further gated into $CD4^+$ and $CD8^+$ subsets. Within the $CD4^+$ population, subsets were identified based on CD45RA and CCR7 expression: naïve ($CD45RA^+CCR7^+$), central memory ($CD45RA^-CCR7^+$), effector memory ($CD45RA^-CCR7^-$), and terminally differentiated effector $CD4^+$ T-cells ($CD45RA^+CCR7^-$). Regulatory $CD4^+$ T-cells were gated as CD127^lowCD25^high within the $CD4^+$ subset. Activated $CD4^+$ T-cells were identified based on CD28 expression. $CD8^+$ T-cells were similarly subdivided into naïve, central memory, and effector populations using CD45RA, CCR7, and CD27 markers.

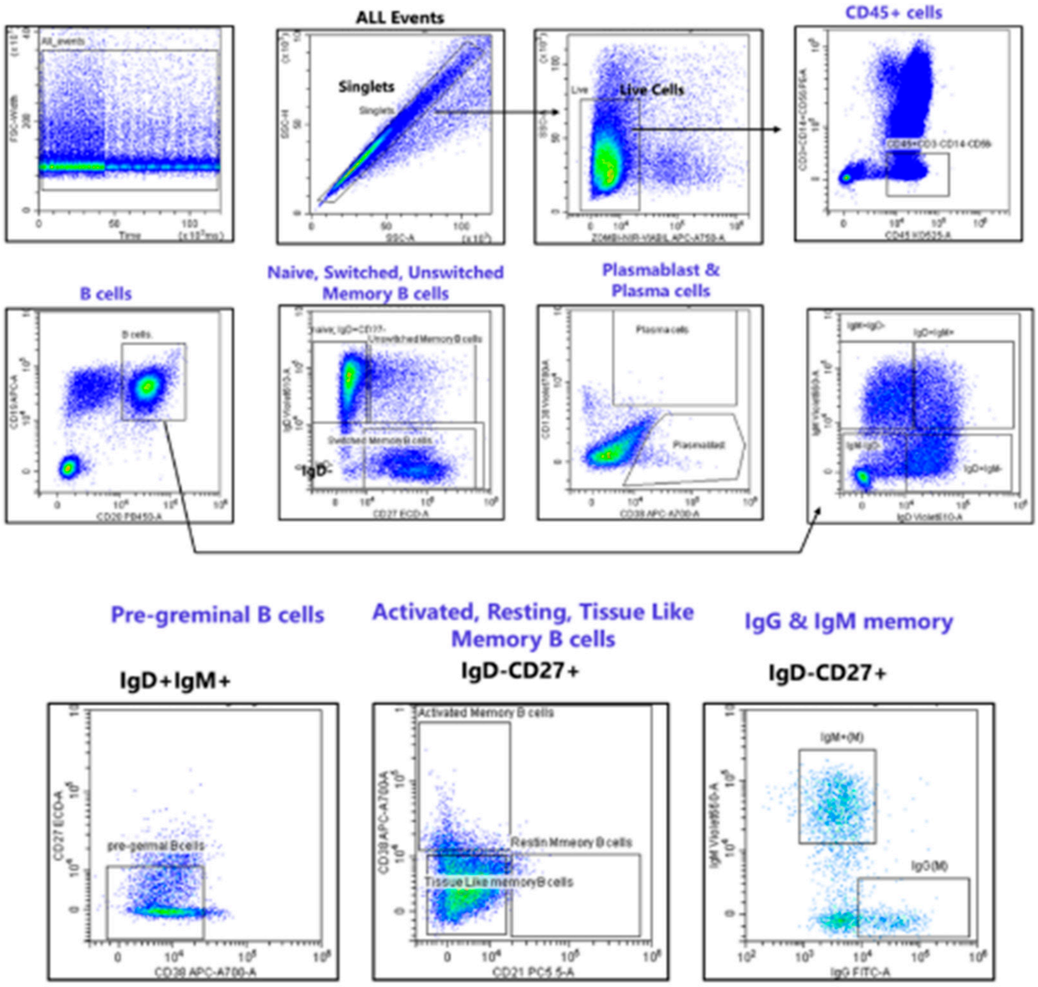

Supplementary Figure S2. Gating strategy for identification of B-cell subpopulations. Flow cytometry gating was performed on live, singlet, CD45^{+} leukocytes. B-cells were identified as CD3^{-}CD14^{-}CD56^{-}CD19^{+} cells. Memory B cell subsets were distinguished based on expression of IgD and CD27: naïve (IgD^{+}CD27^{-}), unswitched memory (IgD^{+}CD27^{+}), switched memory (IgD^{-}CD27^{+}), and double-negative (IgD^{-}CD27^{-}) B-cells. Plasmablasts gated based on CD27^{+}CD38^high expression within the CD19^{+} population and plasma cells were gated based on CD27^{+}CD38^CD138 high expression within the CD19^{+} population. Further classification included pre-germinal B-cells (IgD^{+}IgM^{+}), activated and resting memory B-cells (CD21^{+}CD27^{+}), tissue-like memory B-cells (CD21^{-}CD27^{-}), and isotype-specific memory subsets including IgG^{+} and IgM^{+} memory B-cells (IgD^{-}CD27^{+}IgG^{+}/IgM^{+}).

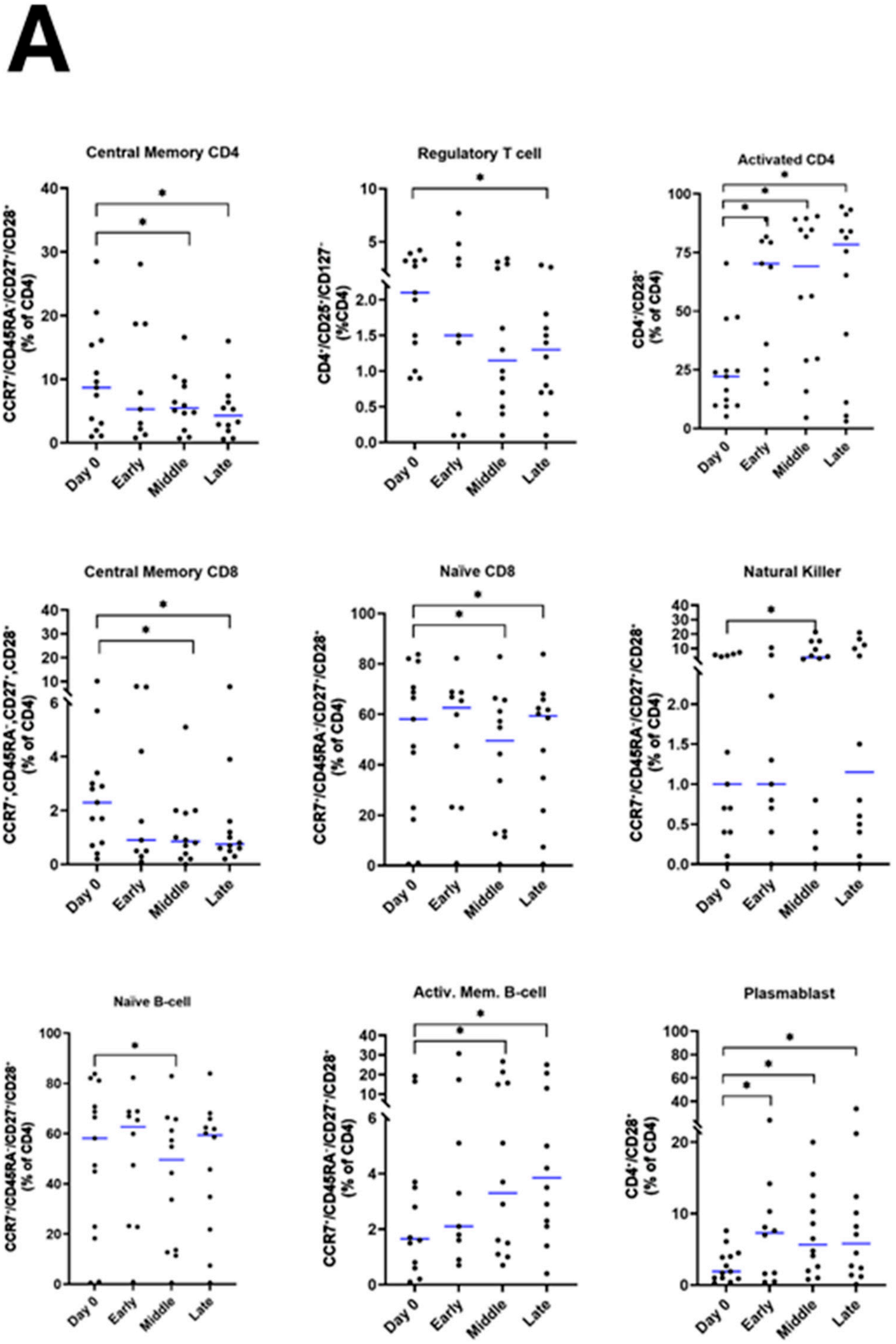
A
Central Memory CD4
Regulatory T cell
Activated CD4
Central Memory CD8
Naïve CD8
Natural Killer
Naive B-cell
Activ. Mem. B-cell
Plasmablast
(% of CD4)
(%CD4)
Day 0
Early
Middle
Late

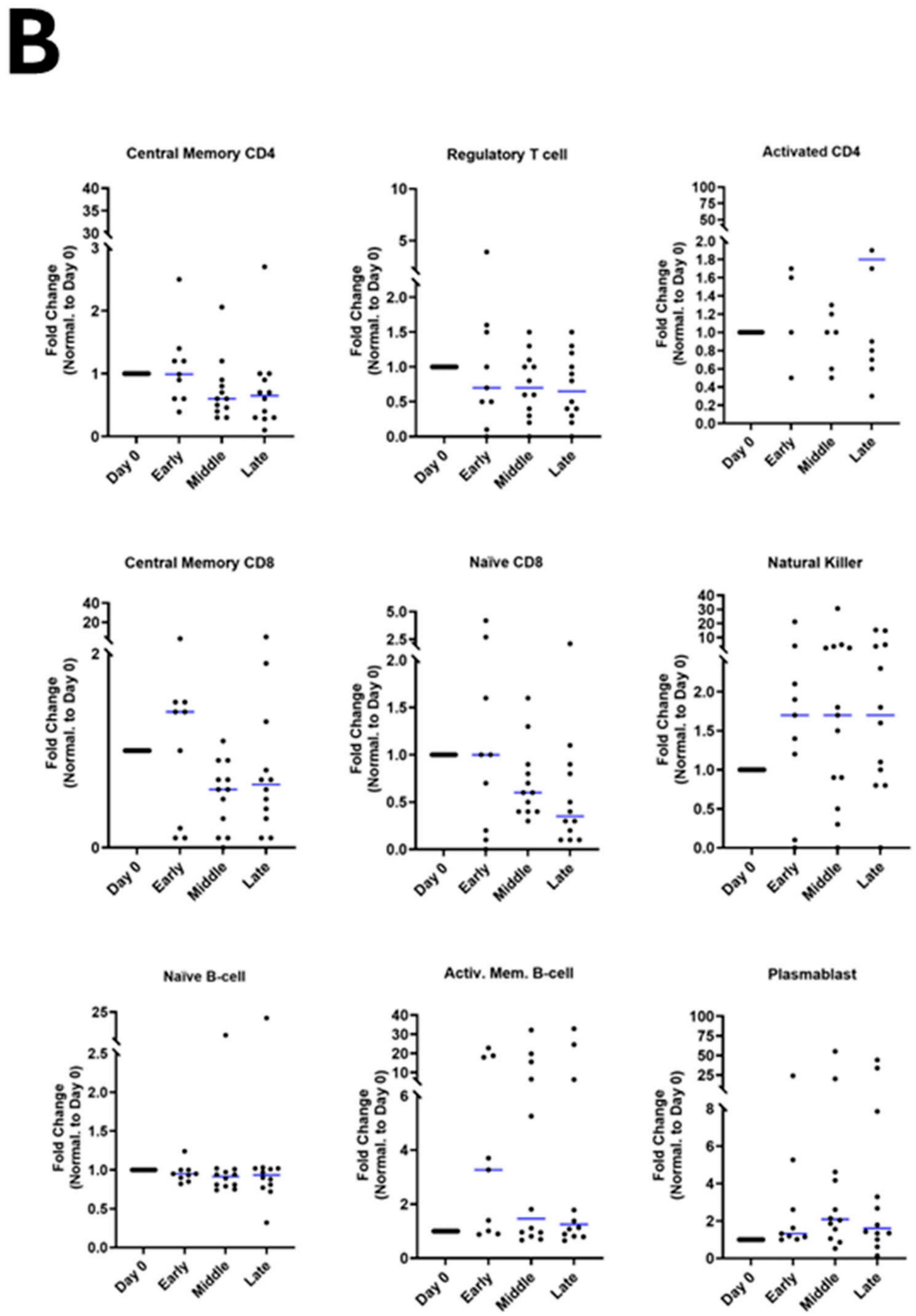

Supplementary Figure S3. Individual patient immune cell responses over time points with significant changes from baseline. A) Scatter plots show absolute values for each patient for immune populations that are statistically significant versus Day 0 (baseline). B) Scatter plots show fold changes for each individual patient (n=13) in selected immune cell populations at Day 0 (baseline), early, middle and late time points. Statistical significance was calculated by Wilcoxon test and it is indicated as follows: *p <0.05; **p < 0.01; ***p < 0.001; ns, not significant.

Supplementary Table S3. The table reports immune populations profiled by flow cytometry that did not show statistically significant differences compared to baseline. For each population, the median fold change over baseline is shown alongside the p-value, which was calculated using absolute values.

	Early/Baseline		Middle/Baseline		Late/Baseline	
	Median Fold Change	p-value	Median Fold Change	p-value	Median Fold Change	p-value
CD45 Lymphocyte	1.1	n.s	1.1	n.s	1.0	n.s
NKT Cells	0.8	n.s	0.7	n.s	0.9	n.s
CD3 T cell	1.5	n.s	1.1	n.s	1.2	n.s
CD4 T cell	0.9	n.s	0.9	n.s	1.0	n.s
CD8 T cell	1.0	n.s	0.9	n.s	1.2	n.s
Effector memory CD4	1.0	n.s	1.1	n.s	1.1	n.s
Terminal Effector Memory CD4	0.6	n.s	0.7	n.s	0.7	n.s
Naïve CD4	0.9	n.s	0.6	n.s	0.5	n.s
Exhausted CD4/PD-1	1.0	n.s	1.0	n.s	0.8	n.s
Activated CD8 Tcell	1.4	n.s	1.2	n.s	1.4	n.s
Effector Memory CD8	0.9	n.s	1.2	n.s	1.5	n.s
Terminal Effector Memory CD8	0.9	n.s	0.9	n.s	0.8	n.s
Exhausted CD8/PD-1	1.0	n.s	0.8	n.s	0.7	n.s
B cells	1.0	n.s	0.9	n.s	1.1	n.s
Memory B cells	1.1	n.s	1.2	n.s	1.2	n.s
Switched Memory B cells	1.0	n.s	1.2	n.s	1.1	n.s
Unswitchd Memory B cells	0.9	n.s	1.0	n.s	1.1	n.s
IgG B cells	0.9	n.s	1.0	n.s	0.9	n.s
IgM B cells	1.0	n.s	1.0	n.s	1.1	n.s
Plasma cells	3.6	n.s	3.0	n.s	3.0	n.s

P-values were calculated based on the percentage frequency of each immune cell subset out of its respective parent population
Statistical significance is indicated as follows:p < 0.05 (*), p < 0.01 (**) and p ≥0.05 (n.s)

Supplementary Table S4. Longitudinal profiling of circulating IgG1 responses in individual patients. Each table summarizes IgG1 levels in serum at middle, late, and chronic time points after ablation. Patients are indicated by letter IDs (rows). Fold changes were calculated relative to baseline levels, and statistical significance is denoted by q-values. Statistically significant upregulation is highlighted in orange, while downregulation is shown in blue. Missing samples are indicated by "/". Statistical significance is indicated as follows: $q < 0.05$ (*), $q < 0.01$ (**), $q < 0.001$ (***), and $q > 0.05$ (ns).

	Biopsy-specific IgG					
	Middle		Late		Chronic	
Patient ID	Fold change	q-value	Fold change	q-value	Fold change	q-value
A2	0.95	ns	0.85	ns	0.90	ns
A3	1.18	ns	**1.96**	*	1.30	ns
A4	**1.94**	***	**1.93**	**	0.60	ns
A9	/	/	0.71	ns	0.68	ns
A11	/	/	0.77	ns	0.87	ns
A17	0.89	ns	0.88	ns	0.99	ns
A18	/	/	/	/	/	/
A25	1.08	ns	**1.41**	**	/	/
A26	0.87	ns	**0.81**	*	1.19	ns
A14	/	/	/	/	/	/
A15	/	/	1.18	ns	/	/

	WT1 IgG					
	Middle		Late		Chronic	
Patient ID	Fold change	q-value	Fold change	q-value	Fold change	q-value
A2	0.91	ns	0.96	ns	0.89	ns
A3	1.06	ns	1.03	ns	1.00	ns
A4	**0.83**	*	**0.89**	*	0.95	ns
A9	0.96	ns	0.91	ns	**0.89**	*
A11	**0.86**	*	**0.88**	*	**0.84**	***
A17	0.96	ns	0.88	ns	1.00	ns
A18	0.96	ns	0.90	ns	0.87	ns
A25	0.93	ns	0.94	ns	/	/
A26	1.03	ns	1.03	ns	**0.89**	*
A14	**0.92**	*	/	/	/	/
A15	/	/	1.00	ns	/	/

	MAGE-A3 IgG					
	Middle		Late		Chronic	
Patient ID	Fold change	q-value	Fold change	q-value	Fold change	q-value
A2	**0.87**	**	**0.94**	*	**0.87**	*
A3	1.07	ns	1.10	ns	1.00	ns
A4	**0.89**	**	**0.91**	*	0.92	ns
A9	0.93	ns	0.91	ns	**0.88**	*
A11	0.97	ns	0.98	ns	0.96	ns
A17	0.93	ns	**0.89**	*	1.06	ns
A18	0.99	ns	0.95	ns	0.97	ns
A25	**0.80**	*	0.84	ns	/	/
A26	0.93	ns	1.00	ns	**0.80**	*
A14	**0.87**	*	/	/	/	/
A15	/	/	1.00	ns	/	/

	MUC1 IgG					
	Middle		Late		Chronic	
Patient ID	Fold change	q-value	Fold change	q-value	Fold change	q-value
A2	**0.84**	*	**0.88**	*	**0.80**	*
A3	**1.10**	*	**1.15**	*	1.11	ns
A4	**0.76**	*	**0.76**	*	0.82	ns
A9	**0.86**	**	**0.84**	*	**0.87**	***
A11	**0.84**	**	0.95	ns	0.92	ns
A17	1.05	ns	1.04	ns	1.13	ns
A18	0.90	ns	**0.83**	*	1.01	ns
A25	0.94	ns	**0.82**	*	/	/
A26	1.00	ns	1.03	ns	**0.81**	*
A14	**0.85**	*	/	/	/	/
A15	/	/	0.99	ns	/	/

	MAGE-A4 IgG					
	Middle		Late		Chronic	
Patient ID	Fold change	q-value	Fold change	q-value	Fold change	q-value
A2	0.86	ns	0.96	ns	0.96	ns
A3	**1.14**	*	**1.17**	*	1.04	ns
A4	0.95	ns	1.01	ns	1.06	ns
A9	0.90	ns	1.05	ns	1.05	ns
A11	1.03	ns	0.92	ns	0.92	ns
A17	0.90	ns	0.91	ns	1.16	ns
A18	0.99	ns	0.94	ns	1.04	ns
A25	0.98	ns	0.98	ns	/	/
A26	1.07	ns	**1.17**	*	0.84	ns
A14	**0.89**	*	/	/	/	/
A15	/	/	0.93	ns	/	/

	NY-ESO-1 IgG					
	Middle		Late		Chronic	
Patient ID	Fold change	q-value	Fold change	q-value	Fold change	q-value
A2	**0.86**	*	0.94	ns	**0.90**	*
A3	1.06	ns	1.04	ns	0.99	ns
A4	**0.89**	*	**0.88**	*	1.01	ns
A9	**0.76**	*	1.05	ns	1.06	ns
A11	**0.89**	*	**0.90**	**	**0.88**	**
A17	0.98	ns	0.94	ns	1.01	ns
A18	0.98	ns	0.96	ns	1.00	ns
A25	**0.84**	*	**0.87**	*	/	/
A26	1.03	ns	1.06	ns	0.90	ns
A14	0.94	ns	/	/	/	/
A15	/	/	0.88	ns	/	/
